# The dose-response relationship between tobacco smoking and the risk of lymphomas: a case-control study

**DOI:** 10.1186/s12885-017-3414-2

**Published:** 2017-06-16

**Authors:** Martina Taborelli, Maurizio Montella, Massimo Libra, Rosamaria Tedeschi, Anna Crispo, Maria Grimaldi, Luigino Dal Maso, Diego Serraino, Jerry Polesel

**Affiliations:** 1 Unit of Cancer Epidemiology, CRO Aviano National Cancer Institute, via Franco Gallini 2, 33081 Aviano, PN Italy; 2 Unit of Epidemiology, National Cancer Institute “G. Pascale” Foundation, via Marino Semmola, 80131 Naples, Italy; 3 Department of Biomedical and Biotechnological Sciences (Biometec), University of Catania, via Androne 83, 95124 Catania, Italy; 4 Unit of Microbiology, Immunology and Virology, via Franco Gallini 2, 33081 Aviano, PN Italy

**Keywords:** Case-control study, Dose-response relationship, Hodgkin lymphoma, Non-Hodgkin lymphoma, Spline models, Tobacco smoking

## Abstract

**Background:**

Previous studies have provided limited support to the association between tobacco smoking and lymphomas with weak evidence of a dose-response relationship.

**Methods:**

We investigated the relationship between tobacco smoking and risk of non-Hodgkin lymphomas (NHL) and Hodgkin lymphomas (HL) through logistic regression spline models. Data were derived from an Italian hospital-based case-control study (1999–2014), which enrolled 571 NHLs, 188 HLs, and 1004 cancer-free controls. Smoking habits and other lifestyle factors were assessed through a validated questionnaire. Odds ratios (OR) and 95% confidence intervals (CI) were estimated by logistic regression, adjusting for potential confounders.

**Results:**

Compared to never smokers, people smoking ≥15 cigarettes/day showed increased risks of both NHL (OR = 1.42, 95% CI: 1.02, 1.97) and HL (OR = 2.47, 95% CI: 1.25, 4.87); the risk was particularly elevated for follicular NHL (OR = 2.43; 95% CI:1.31–4.51) and mixed cellularity HL (OR = 5.60, 95% CI: 1.31, 23.97). No excess risk emerged for former smokers or people smoking <15 cigarettes/day. Spline analyses showed a positive dose-response relationship with significant increases in NHL and HL risks starting from 15 and 21 cigarettes/day, respectively, with the most evident effects for follicular NHL and mixed cellularity HL. Smoking duration was significantly associated with the HL risk only (OR = 2.15, 95% CI: 1.16, 3.99).

**Conclusions:**

These findings support a role of tobacco smoking in the etiology of both NHL and HL, providing evidence of a direct association of risk with smoking intensity.

## Background

In Europe, approximately 93,500 new cases of non-Hodgkin lymphoma (NHL) and 17,500 of Hodgkin lymphoma (HL) were diagnosed in 2012 [1]. When combined, these two lymphoid malignancies represent the eighth most commonly diagnosed cancer in Europe (more than 3% of all new cancer cases), and the sixth in Italy [1].

The etiology of NHL and HL remains poorly understood with just few firmly established risk factors. Immune suppression and viral infections are the most important risk factors for NHL and HL [2]; nonetheless, they are often related to specific histological subtypes [3], accounting for only a small proportion of the overall incidence of lymphomas [4, 5]. Notably, hepatitis C and –to a lesser extent– hepatitis B viruses, have been associated with NHL in several studies conducted in many countries [2], including Italy [6].

Tobacco smoking is a potential risk factor for NHL and HL worth scrutinizing. Several investigations have explored the role of tobacco smoking on the risk of NHL pointing to the etiologic heterogeneity among NHL subtypes [7, 8]. Indeed, it has been consistently shown that smoking may be associated only with certain NHL histological types, particularly follicular lymphoma (FL), with little evidence of a dose-response relationship [7, 9]. Although some inconsistencies exist, results from previous investigations have generally supported a causal association between tobacco smoking and HL, highlighting a direct relationship between a higher number of cigarettes smoked per day and years of smoking and an increased risk of developing HL [8]. Although the evaluation of specific HL subtypes has been limited, most studies have reported that tobacco smoking is associated with an increased risk of mixed cellularity HL [10].

Because of limited results of epidemiological studies on the dose-response relationship between tobacco smoking and risk of lymphoma and its histological subtypes, we conducted a case-control study in three areas of Italy. To provide more accurate risk estimates than categorical analysis we used a flexible approach for the estimation of the dose-response relationship, applying regression spline models.

## Methods

We analyzed data from two consecutive case-control studies on lymphomas, conducted with similar study protocols in two periods, 1999–2002 [11] and 2003–2014 [12].

### First study period, 1999–2002

The study design and findings have been described elsewhere [11, 13, 14]. Briefly, the study conducted between 1999 and 2002 included 231 cases (median age: 59 years) with a new histologically confirmed diagnosis of NHL and 62 with HL (median age: 30 years). All cases were aged ≥18 years and were enrolled in two National Cancer Institutes and general hospital in the province of Pordenone, northeastern Italy, and the town of Naples, southern Italy. Controls were 547 cancer-free inpatients frequency-matched according to center (Pordenone, Naples), gender, and age (in 5-year age groups) based on the distribution of overall study cases, which also included hepatocellular carcinomas (HCCs) [11, 13]. Data from this first study period were published in 2005 in form of odds ratios [11] and later, in 2014, included in a large publication of the InterLymph Consortium [3].

### Second study period, 2003–2014

Between 2003 and 2014, we extended the previous study, focusing only on lymphomas, and maintaining the same study design, inclusion and exclusion criteria, and questionnaire. Cases were patients aged 18–84 years with incident, histologically confirmed diagnosis of NHL (*n* = 353; median age: 56 years) or HL (*n* = 130, median age: 33 years). They were admitted to National Cancer Institutes and general hospitals in the province of Pordenone, northeastern Italy, and the towns of Naples and Catania, southern Italy. Five hundred thirty seven inpatients (median age: 50 years), admitted for a wide spectrum of acute, non-neoplastic conditions to the same hospitals as lymphomas cases, were enrolled as controls. They were frequency-matched by center (Pordenone, Naples, and Catania), gender, and age (in 5-year age groups) based on the distribution of all cases.

### Complete study dataset

The small sample size of cases and controls collected separately in the first and second study periods (1999–2002; 2003–2014) did not allow to adequately address subgroup analyses or interactions [11]. Therefore, data from the two study periods were combined to improve statistical power so that the present analysis included 571 NHL and 188 HL cases with complete information on smoking status and blood samples. All cases were routinely tested for HIV, reporting negative results. Histological records were centrally revised, and lymphomas were classified according to the International Classification of Diseases for Oncology (third edition) [15]. Cancer-free patients admitted to hospitals for at least one of the following conditions were not eligible as controls: a) hematologic, allergic, or autoimmune disorders; b) diseases associated to tobacco consumption, alcohol abuse, or hepatitis viruses infections; c) chronic conditions that might have induce long-term changes in lifestyle habits. However, comorbidity for the above listed diseases was not an exclusion criterion. Overall, controls were admitted for the following reasons: non-traumatic orthopedic diseases (39.4%); acute surgical conditions (20.9%); trauma (20.4%); eye diseases (9.2%); other conditions (10.1%).

The control group for NHL included 1004 inpatients with available blood samples. Since controls were matched also to HCC cases in the period 1999–2002, controls for NHL cases were more likely men and slightly younger than cases. Concerning HL cases, in view of their peculiar age distribution, a set of 188 subjects was selected from the pool of 1004 controls; one control was matched to each HL case according to center, year of enrolment, gender and age.

All study participants signed an informed consent, according to the requirements of the Board of Ethics of each study center, which approved the study.

### Questionnaire

Trained interviewers administered a structured questionnaire to cases and controls during their hospital stay, thus reducing to <5% the refusal rate of both cases and controls. The questionnaire included specific sections to assess information on socio-demographic indicators and tobacco consumption [11]. Information on smoking included smoking status (i.e., never, former, or current smoker), daily number of cigarettes/cigars and grams of pipe-tobacco smoked, age at starting and quitting, and duration of the habit. Smokers were defined as subjects who had smoked at least one cigarette per day for at least 12 months. Former smokers were those who had abstained from cigarette smoking for at least 12 months before the interviews. Considering the low prevalence of cigar and pipe smoking, in our computations, 1 g of pipe-smoked tobacco corresponded to one cigarette, and one cigar to three cigarettes. The validity and reproducibility of questions on self-reported smoking habits in our study population were satisfactory [16].

### Statistical methods

The risk of NHL and HL was estimated through odds ratios (ORs) and corresponding 95% confidence intervals (CIs), calculated by unconditional multiple logistic regression, including gender, age (in quinquennia plus a term for age as a continuous variable), study center, years of education, and place of birth [17]. Additional adjustment for alcohol drinking did not substantially modify risk estimates. Tests for trend were based on the likelihood-ratio test between the models with and without a linear term for each variable of interest. Tests for heterogeneity were computed by comparing the models with and without an interaction term [17].

The dose-response relationship between number of cigarettes/day and risk of NHL and HL was investigated using logistic regression spline models, and the appropriate pointwise CIs were also calculated [18]. Briefly, the logit was estimated through a generalized semi-parametric model where the exposure (i.e., smoking intensity) was included as a smoothly piecewise polynomial of defined degree, with constrains for continuity at each join point. The optimal number of segments was detected putting an increasing number of knots and selecting the best-fitting model, defined as the one minimizing the Akaike Information Criterion [19]. ORs from spline models were estimated adjusting for the same factors as the unconditional multiple logistic regression, and “never smokers” were considered as the reference category. Moreover, to prevent estimates instability in the right tail due to sparse data, subjects who smoked >30 cigarettes/day were excluded: 7 NHL cases (1.6%) and 15 relative controls (2.1%); 9 HL cases (5.5%).

## Results

Table 1 shows the distribution of cases and controls by study center, year of interview, gender, age, place of birth, and years of education. Compared to controls, NHL cases were more likely to be born in southern Italy. No differences emerged between HL cases and matched controls.

**Table 1:** Distribution of cases of non-Hodgkin and Hodgkin lymphoma and controls according to selected characteristics

	Non-Hodgkin lymphoma					Hodgkin lymphoma				
	Controls (*n* = 1004)		Cases (*n* = 571)		*p*-value^a^	Controls (*n* = 188)		Cases (*n* = 188)		*p*-value^a^
	No.	%	No.	%		No.	%	No.	%	
Study center										
Aviano	527	52.5	272	47.6		83	44.1	83	44.1	
Napoli	359	35.8	215	37.7		56	29.8	56	29.8	
Catania	118	11.7	84	14.7		49	26.1	49	26.1	
Year of interview										
1999–2002	504	50.2	228	39.9		62	33.0	63	34.0	
2003–2014	500	49.8	343	60.1		126	67.0	125	66.0	
Gender										
Male	622	62.0	318	55.7		102	54.3	102	54.3	
Female	382	38.0	253	44.3		86	45.7	86	45.7	
Age (years)										
< 30	107	10.7	38	6.7		66	35.1	78	41.5	
30–44	198	19.7	99	17.3		79	42.0	67	35.6	
45–64	366	36.4	260	45.5		35	18.6	35	18.6	
≥ 65	333	33.2	174	30.5		8	4.3	8	4.3	
Place of birth^b^										
North-Center	487	48.6	221	38.8		73	38.8	58	31.0	
South	515	51.4	349	61.2	*p* < 0.01	115	61.2	129	69.0	*p* = 0.13
Education (years)^b^										
< 7	368	36.6	195	34.2		18	9.6	21	11.2	
7–11	300	29.9	186	32.6		73	38.8	64	34.2	
≥ 12	336	33.5	189	33.2	*p* = 0.47	97	51.6	102	54.6	*p* = 0.62

^a^Fisher test

^b^The sum does not add up to the total because of missing values

The association between tobacco smoking and risk of lymphoma is shown in Table 2. Among current smokers, no significant increase in NHL risk emerged, as compared to never smokers. Nevertheless, current heavy smokers (i.e., ≥15 cigarettes/day) showed a higher NHL risk (OR = 1.42, 95% CI: 1.02, 1.97). Although not statistically significant, early age at starting smoking (i.e., <18 years) was associated with an increased NHL risk (OR = 1.36, 95% CI: 0.99, 1.88). A similar pattern of risk emerged for HL, with an increased risk among current smokers who smoked more than 15 cigarettes/day (OR = 2.47, 95% CI: 1.25, 4.87) as compared to never smokers (Table 2). Smoking duration of more than 15 years was also associated with an elevated HL risk (OR = 2.15, 95% CI: 1.16, 3.99). Conversely, among former smokers, smoking intensity, smoking duration, age at starting smoking, and time since quitting were not associated with the risk of either NHL or HL.

**Table 2:** Risk of non-Hodgkin and Hodgkin lymphoma according to smoking habits

	Non-Hodgkin lymphoma					Hodgkin lymphoma				
	Controls (*n* = 1004)		Cases (*n* = 571)		OR (95% CI)	Controls (*n* = 188)		Cases (*n* = 188)		OR (95% CI)
	No.	%	No.	%		No.	%	No.	%	
Smoking status										
Never	426	42.4	239	41.9	1^c^	89	47.3	85	45.2	1^c^
Former	296	29.5	146	25.6	0.91 (0.68–1.20)	33	17.6	23	12.2	0.83 (0.43–1.63)
Current	282	28.1	186	32.6	1.18 (0.91–1.54)	66	35.1	80	42.6	1.50 (0.93–2.44)
Current										
Smoking intensity (cig/day)^a^										
*< 15*	141	14.0	78	13.7	0.99 (0.71–1.39)	40	21.3	36	19.2	1.07 (0.61–1.88)
*≥ 15*	139	13.8	108	18.9	1.42 (1.02–1.97)	26	13.8	43	22.9	2.47 (1.25–4.87)
χ^2^ for trend					*p* = 0.04					*p* = 0.02
Increase of 5 cig/day					1.07 (1.00–1.15)					1.28 (1.11–1.49)
Smoking duration (years)^a, b^										
< 30	131	13.1	80	14.0	1.38 (0.96–1.99)	34	18.1	33	17.6	1.03 (0.55–1.93)
≥ 30	151	15.0	105	18.4	1.04 (0.75–1.44)	32	17.0	47	25.0	2.15 (1.16–3.99)
χ^2^ for trend					*p* = 0.46					*p* = 0.03
Age at starting (years)^a^										
≥ 18	130	13.0	75	13.1	1.00 (0.71–1.41)	24	12.8	25	13.3	1.35 (0.69–2.62)
< 18	151	15.0	110	19.3	1.36 (0.99–1.88)	42	22.3	55	29.3	1.60 (0.93–2.77)
χ^2^ for trend					*p* = 0.07					*p* = 0.10
Former										
Smoking intensity (cig/day)^a^										
*< 15*	139	13.9	69	12.1	0.93 (0.66–1.31)	16	8.5	9	4.8	0.72 (0.29–1.80)
*≥ 15*	156	15.6	77	13.5	0.91 (0.64–1.29)	17	9.0	14	7.5	1.07 (0.46–2.51)
χ^2^ for trend					*p* = 0.42					*p* = 0.49
Increase of 5 cig/day					0.99 (0.92–1.06)					1.02 (0.83–1.24)
Smoking duration (years)^a, b^										
< 30	168	16.7	86	15.1	0.95 (0.69–1.31)	12	6.4	14	7.5	1.56 (0.66–3.72)
≥ 30	127	12.7	60	10.5	0.85 (0.58–1.25)	20	10.6	9	4.8	0.44 (0.17–1.13)
χ^2^ for trend					*p* = 0.34					*p* = 0.83
Age at starting (years)^a^										
≥ 18	155	15.5	78	13.7	0.91 (0.65–1.28)	16	8.6	8	4.3	0.53 (0.19–1.46)
< 18	139	13.9	65	11.4	0.87 (0.61–1.26)	16	8.6	15	8.0	1.17 (0.52–2.62)
χ^2^ for trend					*p* = 0.36					*p* = 0.49
Time since quitting (years)^a^										
≥ 16	153	15.3	76	13.4	0.91 (0.64–1.30)	12	6.5	9	4.8	1.39 (0.47–4.11)
< 16	140	14.0	67	11.8	0.86 (0.60–1.23)	19	10.2	13	7.0	1.10 (0.47–2.53)
χ^2^ for trend					*p* = 0.39					*p* = 0.75

Odds ratios (OR) and 95% confidence intervals (CI) were estimated using unconditional logistic regression model adjusted for gender, age, study center, years of education, and place of birth

^a^The sum does not add up to the total because of missing values. ^b^For HL, the cut-off was set at 15 years

^c^Reference category

In the analysis by NHL histological subtypes (Table 3), only follicular NHL was significantly associated with heavy smoking (OR = 2.43, 95% CI: 1.31, 4.51). Nonetheless, other histological NHL subtypes reported increased risks, but the small sample size did not allow to draw conclusions. Regarding HL, heavy smoking was associated with a significantly increased risk of mixed cellularity HL (OR = 5.60, 95% CI: 1.31, 23.97). Moreover, the ORs were 1.76 (95% CI: 0.78, 3.98) for nodular sclerosis, and 3.22 (95% CI: 1.15, 9.04) for other/NOS subtypes.

**Table 3:** Risk of non-Hodgkin and Hodgkin lymphoma subtypes according to smoking habits

	Smoking habits^a^						χ^2^ for trend
	Never^b^	Current					
		<15 cig/day		≥15 cig/day			
	No.	No.	OR (95% CI)	No.	OR	95% CI	
Non-Hodgkin lymphoma							
Mature B-cell lymphomas	219	67	0.95 (0.67–1.34)	95	1.37 (0.97–1.92)		*p =* 0.09
DLBCL	133	33	0.76 (0.49–1.18)	49	1.09 (0.72–1.66)		*p =* 0.88
Burkitt	5	4	3.07 (0.74–12.81)	4	3.30 (0.71–15.28)		*p =* 0.16
Follicular	39	18	1.31 (0.69–2.47)	25	2.43 (1.31–4.51)		*p <* 0.01
Mantle cell	4	1	0.66 (0.07–6.41)	2	1.04 (0.17–6.50)		*p =* 0.99
Marginal zone	16	3	0.66 (0.18–2.44)	4	1.09 (0.33–3.66)		*p =* 0.80
Lymphoplasmacytic	8	3	1.56 (0.38–6.48)	2	0.87 (0.16–4.86)		*p =* 0.99
SLL/CLL	8	4	1.41 (0.40–4.93)	7	2.47 (0.81–7.59)		*p =* 0.06
Other B-cell lymphomas	6	1	0.45 (0.05–4.15)	2	2.87 (0.41–20.02)		*p =* 0.46
Mature T-cell lymphomas	14	8	1.83 (0.72–4.62)	6	1.72 (0.59–5.03)		*p =* 0.15
Other or NOS	6	3	1.20 (0.75–1.91)	7	2.30 (1.40–3.77)		*p <* 0.01
Hodgkin lymphoma							
Nodular sclerosis	61	28	1.14 (0.61–2.12)	18	1.76 (0.78–3.98)		*p =* 0.23
Mixed cellularity	6	2	1.02 (0.16–6.66)	10	5.60 (1.31–23.97)		*p =* 0.03
Other or NOS	18	6	0.78 (0.27–2.21)	15	3.22 (1.15–9.04)		*p =* 0.06

Odds ratios (OR) and 95% confidence intervals (CI) were estimated using unconditional logistic regression model adjusted for gender, age, study center, years of education, and place of birth

*CLL* Chronic lymphocytic leukemia, *DLBCL* Diffuse large B-cell lymphoma, *NOS* Not otherwise specified, *SLL* Small lymphocytic lymphoma

^a^Former smokers excluded. ^b^Reference category

**Fig. 1:**
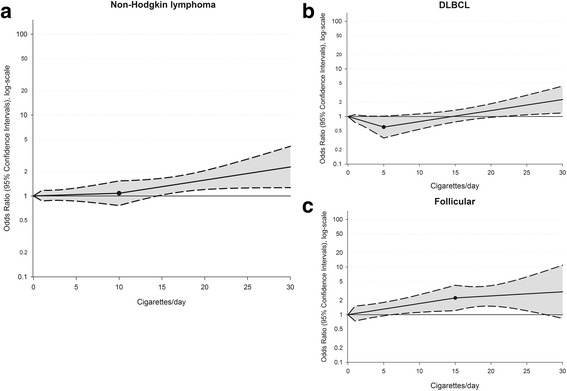
Dose-response relationship between tobacco smoking and the risk of non-Hodgkin lymphoma **a** and its major subtypes: DLBCL **b** and follicular **c**. Odds ratios and 95% confidence intervals were estimated through logistic regression spline models adjusted for gender, age, study center, years of education, and place of birth. Curves are shown for best-fitting splines according to Akaike Information Criterion. The reference category was defined as never smokers. Filled circles show knot location

**Fig. 2:**
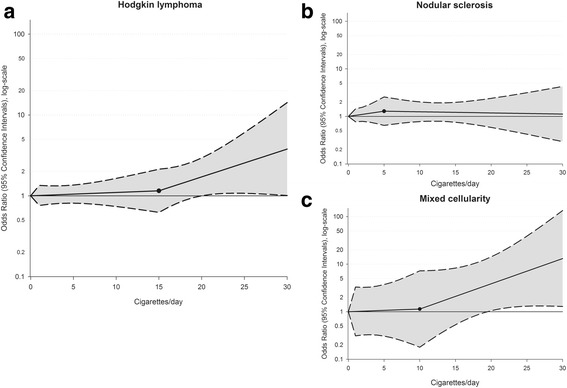
Dose-response relationship between tobacco smoking and the risk of Hodgkin lymphoma **a** and its major subtypes: nodular sclerosis **b** and mixed cellularity **c.** Odds ratios and 95% confidence intervals were estimated through logistic regression spline models adjusted for gender, age, study center, years of education, and place of birth. Curves are shown for best-fitting splines according to Akaike Information Criterion. The reference category was defined as never smokers. Filled circles show knot location

Considering the lack of any association among former smokers, the dose-response relationship between current tobacco smoking and lymphoma risk was investigated through spline models. The shape of best-fitting regression model showed that, for both NHL and HL, the risk steadily increased with increasing number of cigarettes/day above 10 and 15 cigarettes/day, respectively. However, the risk was significant beginning with 15 cigarettes/day (Fig. 1a) for NHL and 21 cigarettes/day for HL (Fig. 2a). Subgroup analyses for the main histological subtypes showed a significant increased risk of follicular NHL (Fig. 1c) after 7 cigarettes/day, whereas the effect was less evident for diffuse large B-cell lymphomas (DLBCL) as the increase in risk turned out to be significant only after 22 cigarettes/day (Fig. 1b). Concerning HL subtypes, the risk of mixed cellularity HL (Fig. 2c) was significantly higher beginning with 20 cigarettes/day. No significant dose-response relationship emerged for nodular sclerosis (Fig. 2b).

Table 4 shows the association between tobacco smoking and NHL risk in separate strata. No heterogeneity in risks emerged across strata of study center, gender, or age.

**Table 4:** Risk of non-Hodgkin lymphoma for smoking habits in selected strata

	Smoking habits^a^							χ^2^ for trend
	Never^b^	Current < 15 cig/day			Current ≥ 15 cig/day			
	Ca:Co	Ca:Co	OR	95% CI	Ca:Co	OR	95% CI	
Study center								
Aviano	133:250	33:68	1.08 (0.70–1.68)		30:45	1.56 (0.95–2.56)		*p =* 0.57
Napoli	72:128	35:53	1.17 (0.71–1.93)		53:60	1.75 (1.07–2.85)		*p =* 0.02
Catania	34:48	10:20	0.52 (0.23–1.19)		25:34	1.03 (0.51–2.06)		*p =* 0.68
						χ^2^ for heterogeneity *p =* 0.39		
Gender								
Male	84:186	39:83	1.14 (0.75–1.75)		80:119	1.68 (1.16–2.43)		*p =* 0.15
Female	155:240	39:58	0.98 (0.63–1.51)		28:20	1.67 (0.91–3.07)		*p =* 0.06
						χ^2^ for heterogeneity *p =* 0.50		
Age (years)								
< 45	66:152	22:63	1.11 (0.70–1.76)		34:49	2.33 (1.39–3.89)		*p =* 0.01
45–64	95:136	41:52	0.96 (0.59–1.57)		57:66	1.33 (0.84–2.10)		*p =* 0.47
≥ 65	78:138	15:26	1.20 (0.57–2.51)		17:24	1.31 (0.61–2.80)		*p =* 0.64
						χ^2^ for heterogeneity *p =* 0.46		

Odds ratios (OR) and 95% Confidence Intervals (CI) were estimated using unconditional logistic regression models adjusted for gender, age, study center, years of education, and place of birth. Ca, cases; Co, controls

^a^Former smokers excluded

^b^Reference category

## Discussion

The findings of this case-control study provided further evidence on the role of smoking in the etiology of both NHL and HL. The study reported positive dose-response relationships based on number of cigarettes smoked per day, highlighting an increase in NHL and HL risks beginning with 15 and 21 cigarettes/day, respectively. Age at smoking initiation was not significantly associated with either NHL or HL risk, whereas smoking duration was found to be significantly associated with the HL risk only.

Results from previous studies on tobacco smoking have not provided a definitive link with NHL [7, 8]. Even if some studies reported a positive dose-response association in terms of smoking intensity [11, 20, 21], most studies have not observed such a relationship [22–25]. Notably, in line with our results, a large pooled analysis of nine case-control studies from the InterLymph Consortium [26] found that heavy smokers had an elevated risk for NHL.

Although several studies have reported a positive association with smoking [27, 28], HL has never been regarded as a smoking-related cancer [7]. In agreement with our findings, a recent meta-analysis [8] evidenced a direct dose-response relationship between higher number of cigarettes smoked per day and number of years smoking, and increased risk of developing HL.

It is worth noting that most of the studies finding positive associations with HL have also observed null or inverse associations with NHL [7, 23, 24]. In the present investigation, the findings regarding HL were super imposable to those obtained for NHL -as no excess risk emerged among former or light smokers as compared to never smokers. Moreover, we found that the risk steadily increased with the number of cigarettes/day for both NHL and HL, yielding analogous effect estimates.

Stratification by subtypes revealed that cigarette smoking may affect risk differently, depending on the lymphoma subtypes. In agreement with our results, FL is the only NHL subtype with a statistically significant association reported consistently [7, 23, 25, 29]. The InterLymph Consortium [9] observed an increased risk of FL among current smokers, although no trend based on smoking intensity was evidenced. On the contrary, two cohort studies [23, 25] found an inverse association between smoking and risk of FL.

The majority of the investigations on smoking and HL lacked sample size and sometimes the histological information needed to distinguish among HL subtypes [23, 24]. Few reports have provided evidence for a role of tobacco smoking in the etiology of mixed cellularity HL [10, 27], in line with our results. Conversely, a European multi-center case-control study [28] showed that current smokers aged ≥35 years had approximately a 2.5-fold higher risk of nodular sclerosis HL than never smokers with a suggestive dose-response relationship.

The association between smoking and lymphoma found in this study is consistent with the evidence that direct carcinogenic effects of smoking are mediated by various chemicals contained in cigarettes such as formaldehyde [30] and benzene [31]. Moreover, smoking may also indirectly affect lymphomagenesis by modulating immune responses [32]. In fact, smoking has been shown to increase lymphocyte subset counts, alter their function, and to down-regulate the activity of natural killer cells and macrophages, thus promoting the pathogenesis of lymphomas [33].

The association of tobacco smoking with HL may be related to an effect of Epstein-Barr virus (EBV) reactivation due to the state of immunodeficiency induced by cigarette smoking [34]. Interestingly, in the present investigation, the association between current smoking and HL was restricted to the mixed cellularity subtype, which is more commonly associated with EBV [2]. Similarly, a pooled analysis from the InterLymph Consortium [10] have reported a higher risk for mixed cellularity and EBV-positive HL among current cigarette smokers in both younger and older individuals and among men. Moreover, a recent survey conducted among young male adults has observed that seroprevalence of EBV was higher among current smokers (93%) than among never smokers (85%) [35].

Some study limitations have to be acknowledged. First, selection and information biases were possible, as in most of hospital-based case-control studies. However, selection bias was limited by paying attention in: a) enrolling cases and controls in the same catchment areas; b) excluding from the control group all patients admitted to hospital for diseases associated to the exposures under study. Information bias, if any, is likely to have had a limited impact on study findings. Indeed, although cases and controls may have recalled their smoking habits differently, awareness of any particular hypothesis about the role of tobacco smoking in lymphomas’ aetiology was limited in our study population at the time the study was conducted. Further, information bias has been minimized by the administration of the questionnaire to both cases and controls under similar conditions. Second, despite the relatively large sample size, the study has still limited power to detect association for specific NHL subtypes and results should be interpreted with caution. The nearly complete participation of identified cases and controls, the satisfactory reproducibility of information on tobacco smoking [16], and the revision of lymphoma diagnosis represent important strengths of our study. Finally, the choice of a more flexible approach for the estimation of the dose-response relationship, such as regression spline models, allowed us to provide more accurate risk estimates than the categorical analysis.

## Conclusions

In conclusion, our results lent additional support to the possibility that tobacco smoking may play a role in the etiology of both NHL and HL, including the HL subtype more commonly associated with EBV. Moreover, the risk of lymphoma appears to be elevated in people reporting a higher number of cigarettes smoked per day. Future studies would greatly benefit from a joint assessment of smoking parameters and biomarkers of infectious agents.

## Abbreviations

CI: Confidence interval
DLBCL: Diffuse large B-cell lymphoma
EBV: Epstein-Barr virus
FL: Follicular lymphoma
HCC: Hepatocellular carcinoma
HL: Hodgkin lymphoma
NHL: Non-Hodgkin lymphoma
OR: Odds ratio
